# Rhythmic expression of an *egr-1* transgene in rats distinguishes two populations of photoreceptor cells in the retinal outer nuclear layer

**Published:** 2008-06-20

**Authors:** Pui-San Man, Tim Evans, David A. Carter

**Affiliations:** School of Biosciences, Cardiff University, Cardiff, United Kingdom

## Abstract

**Purpose:**

Nocturnal rhythms of gene expression in the retina are known to be both darkness- and circadian clock-dependent, but their role and cellular location are not well defined. In the present study we have used a new transgenic rat model (early growth response gene 1-destablized, enhanced green fluorescent protein 2; egr-1-d2EGFP) to investigate the rhythmic regulation of darkness-related gene expression.

**Methods:**

Adult transgenic rats were sampled during the light and dark phases of a standard laboratory lighting schedule. The cellular location of transgene expression in retinal sections was detected either via immunohistochemistry for green fluorescent protein (GFP) or via direct microscopy. The GFP expression pattern was compared to endogenous proteins (Egr-1, melanopsin, rhodopsin) via dual fluorophore immunohistochemistry. Day-night changes in GFP and Egr-1 expression were quantified by western blot analysis of retinal protein extracts.

**Results:**

Nocturnal transgene expression was abundant in the outer nuclear layer (ONL) of the retina, recapitulating expression of the endogenous Egr-1 protein. The transgene provided greatly enhanced visualization of the ONL cellular expression pattern, in part due to cellular filling by GFP molecules that pervade rod photoreceptor cells including inner and outer segments. The transgene was also expressed in isolated (Egr-1-positive) cells of the inner nuclear layer and the ganglion cell layer. In the ONL, a marked day-night rhythm in transgene expression was found to be predominantly within an inner zone of this retinal nuclear layer. This concentration of rhythmic GFP/Egr-1 to the inner ONL was not associated with differential localization of rhodopsin.

**Conclusions:**

Analysis of a novel transgenic rat strain has identified subpopulations of rod photoreceptor cells that differ with respect to rhythmic nocturnal expression of *egr-1*. These studies demonstrate the value of this genetic approach that has provided a model for the functional characterization of retinal rhythms, specifically addressing the role of Egr-1 within nocturnal transcriptional events in a rod photoreceptor population. Because the darkness-dependent induction of Egr-1 is gated by a circadian clock, this model can also provide insights into the cellular mechanisms of circadian regulation in the retina.

## Introduction

Day-night rhythms in retinal protein activity are self-evident given the recurrent changes in extrinsic input to this sensory system. However, the extent to which expression of retinal proteins (and retinal physiology) is regulated by intrinsic circadian pacemakers or clocks is only now being fully appreciated [1-3]. Current research in this field is aimed at identifying the location of retinal circadian clocks and also the targets of this intrinsic mode of temporal regulation.

An emerging role of circadian regulation in the retina (and other organs) is the enablement or “gating” of expression/activity to physiologically appropriate times of the daily cycle [4-6]. For example, retinal synthesis of melatonin is gated to a nocturnal window by circadian control of type-1 adenylyl cyclase gene expression [5]. Previous work in our laboratory in this area has shown that nocturnal induction of transcription factors in the rat retina is circadian-phase dependent and is therefore another target of circadian clock gating [7]. One retinal transcription factor that is regulated in this manner is the zinc-finger protein Egr-1 (Zif268/NGFI-A) [8]. Studies at the mRNA level first showed that *egr-1* expression exhibits a large and prolonged increase following darkness [9]. We have confirmed this darkness-dependent phenomenon at the protein level, and have also shown that the light versus dark amplitude is gated in a circadian manner [7].

Analysis of the darkness-dependent induction of Egr-1 has been limited in our previous studies to the western blot detection of an immunoreactive Egr-1 band in retinal protein extracts. We have now developed a novel transgenic rat model in which *egr-1* expression can be monitored by a destabilized fluorescent reporter vector (d2EGFP) [10]. This experimental model transcends the use of direct immunohistochemical analysis because the fluorescent protein is not confined to the nuclear compartment like Egr-1; cellular filling, particularly in neuronal structures, greatly enhances both visualization and identification of cellular expression patterns [10]. In the present study we have used this new transgenic model to localize the major site of darkness-dependent retinal Egr-1 expression and, in the process, identify rhythmic subpopulations of retinal photoreceptor cells.

## Methods

### Animals and tissue sampling

Animal studies were conducted in accordance with United Kingdom Home Office regulations, and local ethical review. The animals used here were egr-1-d2EGFP transgenic rats [10] in which genomic sequences derived from rat *egr-1* drive expression of the green fluorescent protein (GFP) destabilized derivative, egr-1-d2EGFP (Clontech, Palo Alto, CA) in a Sprague-Dawley background. Egr-1-d2EGFP transgenic rat lines are maintained as hemizygote colonies using PCR-based genotyping as described [10]. In the present study, three lines of egr-1-d2EGFP rats derived from three independent founders were used: Z14, Z16, and Z27B. Adult (3–5 months old), male and female rats of the egr-1-d2EGFP lines were housed in standard laboratory cages, and fed (standard rat chow) and watered ad libitum. Rats were maintained in a 14 h:10 h light–dark (LD) cycle (lights on: 05:00 h), and were killed by cervical dislocation either before, or after the onset of darkness (19:00 h). Whole eyes were fixed in 4% paraformaldehyde in 0.1 M phosphate buffer (24 h, 4 ^o^C) and cryoprotected in 20% sucrose in 0.1 M phosphate buffer at (24h, 4 °C). Following cryoprotection, the tissue was embedded in Cryo-M-Bed (Bright Instrument Company Limited, Huntingdon, UK), and 12 μm transverse sections were cut (Bright OTF cryostat with Magnacut blades; Bright Instrument Company Limited) and mounted on glass slides (SuperFrost Plus, VWR International, Poole, Dorset, UK). Slides were dried briefly and stored at −70 ^o^C before immunohistochemistry.

### Immunohistochemical analysis

Retinal proteins were detected by fluorescence immunohistochemistry as described [10]. Primary antisera used were as follows: anti-Egr-1, C19 (Santa Cruz Biotechnology, Santa Cruz, CA); anti-GFP (rabbit; A11122; Molecular Probes, Eugene, OR); antimelanopsin ( ab19383; Abcam, Cambridge, UK); and antirhodopsin ( R5403; Sigma, St. Louis, MO). Primary antisera were used in combination with the appropriate species-specific, fluorophore-tagged, secondary antisera: Alexa Fluor 488-conjugated goat antirabbit IgG (Molecular Probes Inc.); Alexa Fluor 488-conjugated donkey antimouse IgG (Molecular Probes); Cy3-conjugated donkey antimouse IgG (Jackson Immunoresearch Laboratories Inc., West Grove, PA); and Cy3-conjugated sheep antirabbit IgG (Sigma). Sections were mounted using Vectashield with DAPI (Vector Laboratories, Burlingame, CA) and stored in the dark at 4 °C. The specificity of antigen detection using the GFP antibody in this procedure was examined previously [10] and was confirmed in the present analysis of retinal tissue through omission of primary and secondary antisera, and also through use of nontransgenic tissue. The specificity of Egr-1 detection using the C19 antibody has been confirmed previously [11]. C19 specificity was reconfirmed in the present study using an identical peptide preabsorption protocol, which showed that detection of Egr-1 was not observed following overnight incubation of C19 with an excess of peptide immunogen. The specificity of melanopsin detection using the ab19383 antisera was confirmed through detection of a specific and limited population of retinal cells [12] and also through antibody omission controls. The specificity of rhodopsin detection using the R5403 antibody was confirmed through detection of a highly characteristic abundance of expression in the photoreceptor outer segment (OS) region and through antibody omission controls.

Retinal sections were viewed with either an epifluorescence microscope (Leica DM-LB; Leica Microsystems Imaging Solutions Ltd., Cambridge, UK) or a laser confocal microscope (Leica TCS-SP2-AOBS; Leica Microsystems Imaging Solutions Ltd.). Representative images were obtained with, respectively, a Leica DFC-300FX digital camera and Leica QWin software (V3) or Leica Confocal Software. Images were rotated, cropped, and montaged in Photoshop (CS2; Adobe Systems Inc., San Jose, CA).

### Western blot analysis

Whole cell extracts of dissected retina were prepared as described previously [7]. Briefly, retinal tissue was disrupted in ice cold buffer (20 mM HEPES, pH 7.9; 1.5 mM MgCl_2_; 0.42 mM NaCl; 0.2 mM EDTA; 0.5 mM DTT; 25% glycerol and Sigma protease inhibitor cocktail P8340) using a dounce homogenizer, and protein supernatants were obtained following freeze-thaw and centrifugation. Denatured retinal proteins were resolved on SDS–PAGE gels (16% Tris-Glycine Novex gels; Invitrogen, Carlsbad, CA), and specific protein bands were detected by western blotting as described [7,13]. The following primary antisera were used: monoclonal anti-GFP (8362–1; Becton Dickinson, Palo Alto, CA); anti-Egr-1 (4152; Cell Signaling Technology, Danvers, MA); and anti-GAPDH (ab9485; Abcam). The specificity of d2EGFP detection on western blots using the 8362–1 antisera has been confirmed in our previous studies [13]. The specificity of Egr-1 detection using the 4152 antibody was confirmed on parallel western blots using the C19 Egr-1 antibody (Santa Cruz), which we have previously characterized [11]. However, we noted that in addition to a specific Egr-1 band, the 4152 antibody also detected another, abundant, nonspecific retinal protein band, indicating that this antibody may not be suitable for immunohistochemistry. The relative levels of protein bands were determined by densitometry (Imagequant^TM^ 3.0; GE Healthcare UK, Little Chalfont, Bucks, UK) and corrected for extraction and loading variation against the equivalent GAPDH band. A quantitative estimate of L versus D levels of d2EGFP and Egr-1 was obtained by comparing the levels in six animals of the Z16 line.

### Direct fluorescence analysis

For direct (without immunohistochemical procedure) analysis of retinal fluorescence in fixed tissue sections, slides were removed from −70 °C storage, rinsed in 0.01 M phosphate buffered saline (PBS), and mounted in Vectashield with DAPI (Vector). For direct analysis of retinal fluorescence in fresh tissue, dissected retinal sheets were floated onto a pool of PBS in the center of a microscope slide and coverslipped, resulting in cellular dispersal. Observations were made on parallel-processed transgenic and nontransgenic tissue.

## Results

**Figure 1:**
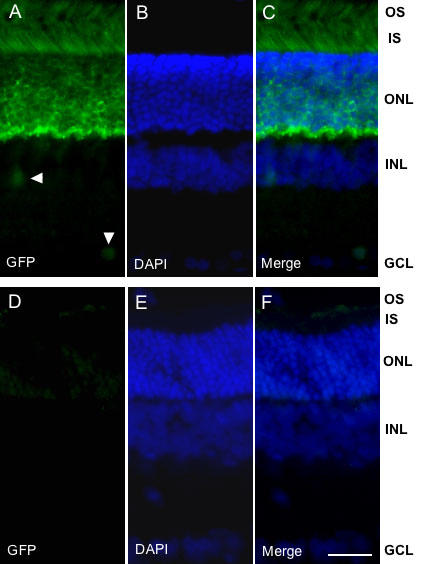
Expression of the egr-1-d2EGFP transgene in adult rat retina. Representative fluorescence microscopic images of transverse sections of adult rat retina showing detection of total cells (DAPI staining) and d2EGFP immunoreactivity (green fluorescent protein, GFP). Animals were killed at 24:00 h. Panels **A-C** show egr-1-d2EGFP transgenic rats and panels **D-F** show wild-type rats. Note detection of abundant GFP immunoreactivity in the outer nuclear layer (ONL), photoreceptor inner segment (IS), and photoreceptor outer segment (OS) of the transgenic rat only. In addition, single GFP-positive inner nuclear layer (INL; horizontal arrowhead) and ganglion cell layer (GCL, vertical arrowhead) cells are indicated in **A**. The scale bar represents 20 μm.

The retina of transgenic (egr-1-d2EGFP) rats exhibited abundant transgene expression (Figure 1). Initial observations were made on tissue obtained during the dark phase because our previous studies have demonstrated an upregulation of endogenous Egr-1 during this part of the daily cycle [7]. GFP expression was largely confined to photoreceptor cells that exhibit a characteristic honeycomb-like appearance in the outer nuclear layer (ONL) and a characteristic feather-like appearance in the photoreceptor units, both inner segment (IS) and OS. Within the ONL, GFP appeared to be confined to the extra-nuclear cellular compartment, a finding that was confirmed by scanning through optical sections on a confocal microscope (not shown). Isolated GFP-positive cells were also observed in the inner nuclear layer (INL) and ganglion cell layer (GCL; Figure 1A). This pattern of transgene expression was maintained in three independent transgenic lines: Z14 (not shown), Z16, and Z27B. GFP-like background fluorescence was minimal in wild-type littermates (Figure 1D-F).

**Figure 2:**
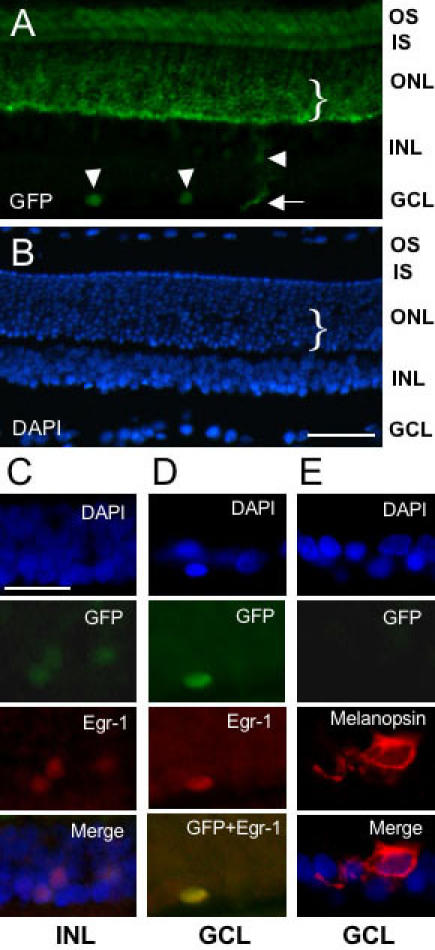
Relative abundance of transgene expression across the retinal nuclear layers and co-localization with Egr-1. Representative fluorescence microscopic images of transverse sections of adult rat retina showing detection of total cells (DAPI staining), and immunoreactivities for d2EGFP (green fluorescent protein, GFP), Egr-1 and melanopsin. Animals were killed at 24:00 h. **A, B**: Extensive view of transgenic rat retina illustrating the abundant and uniform expression of GFP across the outer nuclear layer (ONL), photoreceptor inner segment (IS), and photoreceptor outer segment (OS) relative to the lower abundance and isolated cellular expression within the inner nuclear layer (INL) and ganglion cell layer (GCL). Vertical arrowheads indicate GFP-positive ganglion cells; horizontal arrowhead denotes a GFP-positive INL cell; horizontal arrow marks a GFP-positive process running up from the GCL. An inner zone of the ONL is indicated by a bracket (}); note the relative abundance of GFP within this inner ONL zone. **C:** Co-expression of GFP and Egr-1 within three cells of the INL. Note that the low level of GFP is not detectable in the merged image. **D:** Co-localization of GFP and Egr-1 within a single cell of the GCL. **E:** Absence of GFP within a giant melanopsin-positive cell of the GCL. The scale bar represents 50 μm in **A,B** and 20 μm in **C-E**.

Abundant photoreceptor cell transgene expression was observed across the length of the retina with no clear central-peripheral variation (Figure 2A). However, a marked inner ONL-outer ONL variation was consistently observed with markedly higher levels of GFP within an inner region or zone of the ONL (see Figure 2A,B). This finding was studied further (see results below). Figure 2 also illustrates characterization of the INL and GCL transgene expression. In the INL, GFP was most commonly detected in (presumed according to previous findings that are detailed in [14]) amacrine cells of the inner INL, where it was shown to be co-localized with endogenous Egr-1 Figure 2C). In the GCL, GFP was observed in a minor population of cells where it also co-localized with Egr-1 (Figure 2D). We investigated the possibility that this minor population of GCL GFP-positive cells was representative of the melanopsin-expressing retinal ganglion cells (Figure 2E). However, these giant melanopsin-positive cells [12] appeared to form a separate minor GCL population because we observed no co-localization of melanopsin and GFP (Figure 2E).

**Figure 3:**
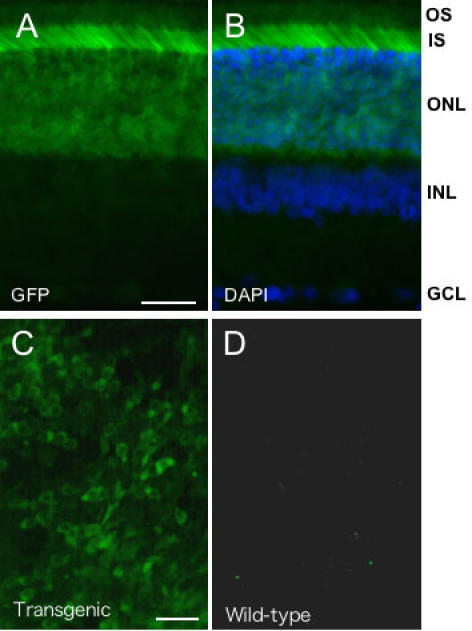
Direct observation of d2EGFP fluorescence in retina. Representative fluorescence microscopic images of adult rat retina sampled at 24:00 h. **A**: Detection of d2EGFP fluorescence in transverse retinal sections. Note the abundance of ONL and IS expression as localized in (**B**) by the corresponding DAPI-merged image. **C**: Detection of d2EGFP fluorescence in flat-mounted freshly dissected retinal tissue from transgenic, but not wild-type animals (**D**). Bars=20 μm. Abbreviations: OS, photoreceptor outer segment; IS, photoreceptor inner segment; ONL, outer nuclear layer; INL, inner nuclear layer; GCL, ganglion cell layer.

Following the initial confirmation and characterization of retinal transgene expression by immunohistochemical detection of d2EGFP, we next showed that GFP fluorescence could be observed by direct microscopy (Figure 3). First, we showed that GFP fluorescence was maintained in paraformaldehyde-fixed sections that had been stored at −70 ^o^C (Figure 3A,B). This analysis confirmed the results of our immunohistochemical analyses (Figure 1 and Figure 2), showing that GFP was primarily active in the ONL. Additional fluorescence was also detectable in the INL and GCL but this activity was less easily defined compared to that obtained by immunohistochemical detection. Currently, we have maintained retinal sections at −70 ^o^C for several months with retention of GFP fluorescence. Second, we have shown that GFP-fluorescent cells can be detected in freshly dissected retinal tissue (Figure 3C,D). GFP fluorescence was maintained at levels above that of wild-type tissue in transgenic tissue stored under coverslips at 4 ^o^C for three days.

**Figure 4:**
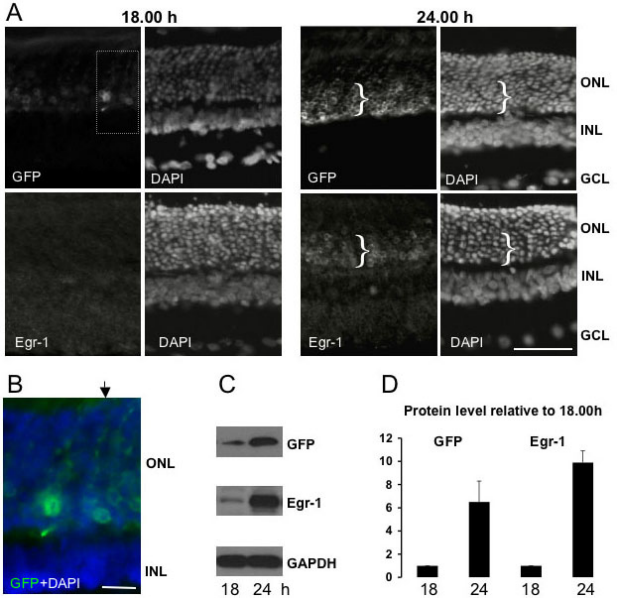
Nocturnal induction of the egr-1-d2EGFP transgene. **A**: Representative fluorescence microscopic images of transverse sections of adult rat retina showing detection of total cells (DAPI staining), and immunoreactivities for d2EGFP (green fluorescent protein, GFP) and Egr-1. Animals were killed either before (18:00 h) or after (24:00 h) the onset of darkness. Note the marked increase in ONL GFP and Egr-1 at 24:00 h and the relative abundance of expressing cells within the inner zone of the ONL, which is indicated by a bracket (}). Bar=50 μm. **B**: Detail from **A** (boxed) showing how the minimal ONL expression of GFP at 18:00 h permits detection of varicosities within ONL fibers (one example of a track of varicosities indicated by arrow). Bar=10 μm. **C**: Representative images of western blots of retinal protein extracts showing an increase in levels of both GFP and Egr-1 at 24:00 h relative to 18:00 h. **D**: Relative levels of GFP and Egr-1 proteins at 18:00 h and 24:00 h detected as in **C**. Values are provided as mean±S.E.M. and are calculated as fold-change relative to the 18.00h level following correction against GAPDH levels. At each time point 3 rats were sampled Abbreviations: ONL, outer nuclear layer; INL, inner nuclear layer; GCL, ganglion cell layer.

Our previous studies of retinal Egr-1 expression by western blot analysis revealed a darkness-dependent, circadian clock-gated, nocturnal upregulation of Egr-1 [7]. In the current study of the new egr-1-d2EGFP transgenic rat line, we therefore sought to confirm whether this aspect of *egr-1* regulation was retained in the transgene. Comparison of retinal GFP expression in animals killed either before (18:00 h) or after (24:00 h) the onset of darkness showed that the level of GFP was markedly reduced at 18:00 h, with clearly detectable ONL cells present only within the inner zone of the ONL (Figure 4A). Minimal ONL GFP expression at this time point revealed the presence of GFP-positive fiber tracts that traversed the ONL and were punctuated with GFP-filled varicosities (Figure 4B). Parallel immunohistochemical analyses with an Egr-1 antibody confirmed nocturnal Egr-1 induction in the ONL (Figure 4A), and furthermore showed that Egr-1 immunoreactivity, like GFP, was markedly more abundant within the inner zone of the ONL (Figure 4A). In this region it was noted that Egr-1 immunoreactivity was confined to the nuclear periphery, as observed for other photoreceptor transcription factor proteins (Otx2 [15]). Despite the presence of an observable increase in Egr-1 immunoreactivity at 24:00 h, Egr-1 detection in the rat retina did not achieve optimal technical resolution because of high levels of background fluorescence. Nevertheless, this analysis was sufficient to confirm that rhythmic expression of the transgene product largely mirrored that of the endogenous protein. A marked difference, however, is that the d2EGFP protein, unlike Egr-1, is not restricted to the nuclear region and is transported to all cellular compartments filling the photoreceptor segments.

Nocturnal upregulation of the transgene was also confirmed by western blot analysis (Figure 4C). The level of a specific d2EGFP immunoreactive band (31 kDa) in whole retinal extracts was found to be markedly higher at 24:00 h compared with the level at 18:00 h, a finding that was confirmed in a total of three animals/time point (Figure 4D). Parallel western blots on the same retinal extracts confirmed that the levels of a specific Egr-1 immunoreactive band (75 kDa) were similarly upregulated during darkness (Figure 4C,D), confirming previous findings [7].

**Figure 5:**
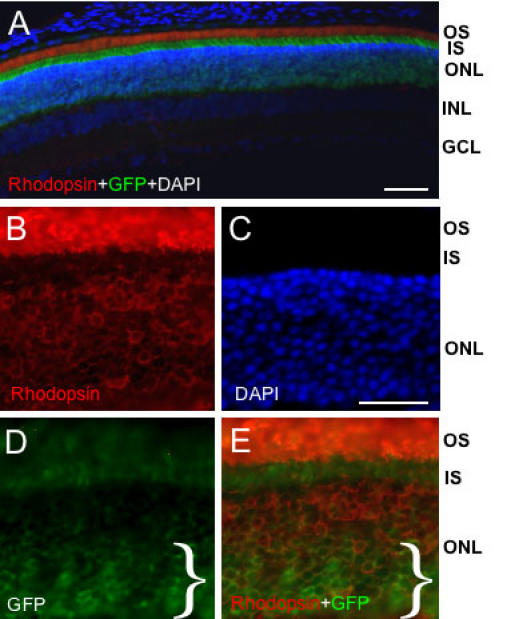
Distribution of rhodopsin relative to egr-1-d2EGFP transgene expression. Representative fluorescence microscopic images of transverse sections of adult rat retina showing detection of total cells (DAPI staining), and immunoreactivities for d2EGFP (green fluorescent protein, GFP) and rhodopsin. Animals were killed at 24:00 h. **A**: Low magnification image showing the massive abundance of rhodopsin protein in the outer segment (OS) region that obscures visualization of (OS) GFP. Note that exposure of the “red” fluorescence signal is minimized in this image (relative to **B** and **E**) such that the much lower level of rhodopsin fluorescence in the ONL is not detectable. Bar=50 μm. **B**-**E**. Relative expression of rhodopsin and GFP across the ONL; note the relative abundance of GFP in the inner zone of the ONL, which is indicated by a bracket (}), whereas rhodopsin is expressed at similar abundance across the extent of the ONL. Bar=20 μm. Abbreviations: OS, photoreceptor outer segment; IS, photoreceptor inner segment; ONL, outer nuclear layer; INL, inner nuclear layer; GCL, ganglion cell layer.

Our novel finding that rhythmic, darkness-dependent *egr-1* transgene and Egr-1 protein expression is concentrated in an inner zone of the ONL indicates that this zone comprises a specific subpopulation of ONL cells. To investigate this inference further, we compared expression of the transgene with that of the rod photoreceptor molecule, rhodopsin. First we confirmed that the rhodopsin antibody detected a massive abundance of this receptor in the photoreceptor OS layer (Figure 5A). The level of OS rhodopsin expression is so high that it entirely obscures GFP expression within the OS layer, even when fluorescence detection of rhodopsin is minimized (see Figure 5A). Higher resolution analysis of rhodopsin expression in the ONL confirmed that this protein is expressed largely uniformly across the ONL (Figure 5B); this is consistent with the known organization of the rodent retina that exhibits minimal outer zone of the ONL containing cone photoreceptors expression (e.g., see [16]). Consequently, when rhodopsin and GFP images are merged (Figure 5E) it is revealed that rhodopsin-positive cells fall into two distinct populations: an inner zone that is highly co-localized with GFP-positive cells and an outer zone where GFP expression is absent or minimal. Therefore GFP/Egr-1 is rhythmically expressed in a subpopulation of rod photoreceptor cells.

## Discussion

### The egr-1-d2EGFP transgene recapitulates retinal expression of egr-1

The present study showed the retina to be one of the major sites of expression in our new egr-1-d2EGFP transgenic rat model. Initially surprising to us, this result is entirely explicable due to the uniform nature of retinal organization and the phenomenon of cellular filling by GFP protein; GFP, unlike Egr-1, is not restricted to the nucleus and is consequently transported throughout the cell, filling the extended photoreceptor elements. Robust and extensive expression of a destabilized fluorescent protein in rat photoreceptors may provide for several downstream applications (see next section). The observed pattern of transgene expression, primarily within the ONL, is in accord both with previous studies that have localized darkness-related transcription factor mRNA expression to this retinal layer [17]and also with the localization of endogenous Egr-1 (present study). Additional sites of transgene expression within subpopulations of INL (presumed amacrine) and GCL cells are also in accord with previous studies that have demonstrated Egr-1 expression within these cell groups (e.g., [14]). The relatively low abundance and occurrence of INL and GCL expression would appear to relate to sampling time because it has been demonstrated that in the mouse retina, at least, relatively few INL and GCL Egr-1-positive are observed at time points beyond 4 h after lights-on [14]. Nevertheless, transgene expression in these minor cellular populations (that co-localizes with Egr-1) together with the abundant (darkness-dependent) ONL expression strongly indicates that the *egr-1* genomic sequences present in our transgene are sufficient to direct a high fidelity level of cell-specific and inducible expression. This conclusion, derived from retinal analysis, is also supported by our previous studies on this transgenic model that investigated postnatal brain development and glial cell expression [10,18].

### Identification of rhythmically distinct outer nuclear layer zones

A second unexpected and interesting finding of the present study, revealed due to the clarity of transgene expression, is the presence of inner and outer zones within the ONL that are distinct with respect to rhythmic GFP expression. These anatomic zones are not artifacts of transgene integration because they were observed in three independent transgenic lines, and moreover were confirmed, albeit at lower resolution, with Egr-1 immunohistochemistry. Through comparison with rhodopsin immunoreactivity it was also shown that the inner and outer zones of GFP expression represent two subpopulations of rod photoreceptor cells. Differential gene expression across the ONL rod population has not been well documented. Some examples are apparent in the in the data of Blackshaw et al. [19]. In the latter study, in situ hybridization analysis revealed differential expression of mRNAs across the ONL; for example, gene 29794 (Y box bp3) is expressed uniformly across the ONL layer, whereas gene 70121 (EST/novel CaBP) is restricted to an inner region of the ONL. Recently, another study demonstrated differential ONL expression of the chromatin protein Hmgb1 [20]. The latter finding is of note because differential expression of this nonhistone “architectural” chromatin protein may influence the expression of many photoreceptor genes. Further studies are required, first to broaden our knowledge of differential ONL gene expression, and second to investigate how these patterns of expression are influenced by the circadian clock.

Our previous study [7] demonstrated that the darkness-dependent induction of Egr-1 in the rat retina is gated in a circadian manner but it is not known whether this temporal regulation is directly mediated by an intrinsic retinal circadian clock. Recent studies in the rat have localized a self-sustaining circadian pacemaker to the photoreceptor (ONL) layer [21], although it is clear that additional retinal circadian pacemakers exist in other (non-ONL) retinal cell groups in mammals [22,23]. It is currently not known whether there are subpopulations of “clock” and “non-clock” cells within the ONL, nor whether such an organization could mediate, to some extent, the differential rhythmic expression of ONL GFP/Egr-1 observed in the present study. Another identified circadian clock output gene in the ONL is *AA-NAT*, which encodes the penultimate enzyme in the melatonin synthesis pathway, a neurohormone that is produced in both the pineal gland and retina [24]. Previous studies have provided evidence of a rhythm in *AA-NAT* mRNA expression that is restricted to the outer zone of the ONL [25,26], although it should be noted that other studies have questioned this restriction [27]. Further analysis of these clock-impinged ONL genes should be of value in uncovering any inner/outer zone organization of the ONL circadian clock.

### The functional role of Egr-1 in rod cells

In addition to questioning the mechanisms that regulate rhythmicity of Egr-1 in the ONL, the present study has also drawn attention to the extensive expression of darkness-related Egr-1 and its potential role in retinal function. A dramatic induction of *egr-1* following the onset of darkness was first demonstrated in the mouse [9] and is therefore conserved across rodent species. Our previous work [7] has shown that although the nocturnal induction of Egr-1 in the rat retina is “gated” in a circadian manner, the magnitude of gene induction is largely darkness-dependent, and would therefore appear to be an adaptive correlate of this environmental state. Consequently, Egr-1 could not be considered a directly “clock-controlled gene” in this system. The physiologic role of egr-1 within photoreceptor cells of the ONL remains unknown and must be considered separately from a distinct role within light and image processing, for example in a smaller population of amacrine cells of the INL (e.g., [14]). The extensive expression of Egr-1 along the ONL suggests a general function—for example, within the renewal of outer segment disks that is rhythmically organized in rod cells [28]. Egr-1 exerts multiple actions in a diversity of central and peripheral systems [10,29]. A previous study has shown that the central (suprachiasmatic nucleus-related) circadian system is functionally intact in *egr-1* knockout mice [30], but so far as we are aware, retinal physiology has not been examined in these mice.

The novel transgenic rat model of retinal gene expression that is described here will be valuable in future studies of Egr-1 and also other aspects of photoreceptor biology. Our model is useful because it recapitulates both anatomic and temporal specificity of expression. Temporal specificity is largely maintained because we have used a destabilized fluorescent protein (d2EGFP) that incorporates a PEST sequence (sequence incorporating a high concentration of amino acids proline (P), glutamic acid (E), serine (S) and threonine (T)) derived from the ornithine decarboxylase protein, targeting this chimeric protein for rapid degradation. The resultant 2 h half-life of d2EGFP is similar to that of Egr-1 [31]. The dynamics of GFP expression that we have monitored in the present study do not exactly match that of the endogenous Egr-1 protein—for example GFP-positive cells are detected in the ONL at 18:00 h whereas Egr-1 is not, at least using the current detection protocol. However, the upregulation at 24:00 h, centered in the inner zone of the ONL, is similar for the two proteins (Figure 4D). Temporal differences in apparent expression probably result from a combination of two main factors: 1) extensive extranuclear GFP expression that expands apparent expression and provides a range of different cellular environments for degradation; and 2) the suboptimal detection of Egr-1 (in the ONL) using the currently available reagents. An aspect of retinal pathology that may benefit from application of the egr-1-d2EGFP model is the study of light-induced retinopathy (e.g., [32]). We have observed (unpublished results) massive ONL cell loss in rats exposed to continuous lighting for a period of weeks, a pathological phenomenon that was directly monitored by GFP fluorescence detection. The presence of an inherently fluorescent molecule in photoreceptor cell populations can also be applied to cell sorting technologies including FACS; the use of RNA amplification should make it possible to derive cellular transcriptomes from sorted cell populations [33]. With respect to the rhythm of Egr-1 studied here, these approaches may lead to the identification of Egr-1 target genes in photoreceptors, and ultimately characterization of Egr-1 function in photoreceptors.

In conclusion, we have used a novel transgenic rat model to provide insights into the darkness-related expression of the transcription factor Egr-1 in photoreceptor cells of the rat ONL. Our findings, together with those of another recent study [20], have provided evidence of gene expression variation between different subpopulations of rod photoreceptor cells and also of differences in rhythmic expression in these populations. Future studies of the retinal transcriptome and its regulation by circadian clock mechanisms should take account of these variations in cellular phenotype.
